# Electrowriting of SU-8 Microfibers

**DOI:** 10.3390/polym16121630

**Published:** 2024-06-08

**Authors:** Diego Armando Sandoval Salaiza, Nico Valsangiacomo, Niyazi Ulas Dinç, Mustafa Yildirim, Jorge Madrid-Wolff, Arnaud Bertsch, Sebastien Jiguet, Paul D. Dalton, Juergen Brugger, Christophe Moser

**Affiliations:** 1 Laboratory of Applied Photonics Devices, Ecole Polytechnique Fédérale de Lausanne (EPFL), 1015 Lausanne, Switzerland; diego.sandovalsalaiza@epfl.ch (D.A.S.S.); nico.valsagiancomo@epfl.ch (N.V.); niyazi.dinc@epfl.ch (N.U.D.); mustafa.yildirim@epfl.ch (M.Y.); jorge.madridwolff@epfl.ch (J.M.-W.); 2 Microsystems Laboratory, Ecole Polytechnique Fédérale de Lausanne (EPFL), 1015 Lausanne, Switzerland; arnaud.bertsch@epfl.ch (A.B.); sebastien.jiguet@epfl.ch (S.J.); juergen.brugger@epfl.ch (J.B.); 3 Phil and Penny Knight Campus for Accelerating Scientific Impact, University of Oregon, 1505 Franklin Boulevard, Eugene, OR 97403, USA; pdalton@uoregon.edu

**Keywords:** melt electrowriting, photonic waveguides, additive manufacturing

## Abstract

As microfiber-based additive manufacturing (AM) technologies, melt electrowriting (MEW) and solution electrowriting (SEW) have demonstrated efficacy with more biomedically relevant materials. By processing SU-8 resin using MEW and SEW techniques, a material with substantially different mechanical, thermal, and optical properties than that typically processed is introduced. SU-8 polymer is temperature sensitive and requires the devising of a specific heating protocol to be properly processed. Smooth-surfaced microfibers resulted from MEW of SU8 for a short period (from 30 to 90 min), which provides the greatest control and, thus, reproducibility of the printed microfibers. This investigation explores various parameters influencing the electrowriting process, printing conditions, and post-processing to optimize the fabrication of intricate 3D structures. This work demonstrates the controlled generation of straight filaments and complex multi-layered architectures, which were characterized by brightfield, darkfield, and scanning electron microscopy (SEM). This research opens new avenues for the design and development of 3D-printed photonic systems by leveraging the properties of SU-8 after both MEW and SEW processing.

## 1. Introduction

Additive manufacturing (AM) has enabled many tools for applications across fields such as tissue engineering [1], photonics [2], and electronics [3]. An emerging subset of AM techniques is electrohydrodynamic processing (EHD) technologies, which include melt and solution electrowriting (MEW [4] and SEW [5]). These technologies are especially adept at fabricating high-porosity materials from ultrafine- and micro-fibers [4,6]. MEW and SEW have similar configurations as traditional electrospinning [7,8,9], which has found widespread applications, from filtration systems to biomedical devices [10]. SEW and MEW are amenable to following AM principles, with controlled deposition of fibers, facilitating the construction of more complex, 3D multi-layered architectures [11]. Prior to performing complex printing, however, the operational parameters need to be determined. Process parameters that affect stability include temperature, applied voltage, mass flow through the nozzle, and collector speed, while the rheological behavior of the solution or melt can change due to physical or chemical changes that can result with time. Such is the case with SU-8, which is a thermoset material that crosslinks with applied heat, which is one of its main features as it alters its mechanical and rheological properties [12,13,14]. For adapting new polymers to MEW, these need to be determined before the creation of detailed and application-specific constructs [15,16].

The primary distinction between MEW and SEW lies in their material processing conditions. MEW utilizes thermoplastic polymers, heated beyond their melting points, to form fibers through rapid cooling upon extrusion [17,18]. This method, free from solvent use, is particularly suited for biomedical applications, where solvent residues might compromise biocompatibility. Conversely, SEW offers a more flexible approach by employing solutions of polymers in suitable solvents, thus broadening the spectrum of processable materials to use, as well as thermoset or heat-sensitive materials. However, this method adds complexity, introducing additional variables such as charge distribution and solvent evaporations leading to solvent choice, which significantly influences the process outcomes [10,19]. In this study, we explore the use of SU-8, a versatile epoxy-based material widely used in microfabrication due to its excellent mechanical, thermal, and chemical stability and its capacity for forming high aspect ratio structures through lithography [20,21,22] for MEW and SEW. Despite its prevalent use in photolithography for the fabrication of 2D structures (see this Special Issue for a thorough review of different non-AM SU-8 processing routes [23]), the potential of SU-8 in creating 3D structures through AM techniques remains largely untapped. This study assesses the feasibility and effectiveness of employing MEW and SEW for fabricating 3D SU-8 structures, targeting applications in photonic and waveguiding systems [24,25] where SU-8’s inherent properties could be highly beneficial [26,27,28,29].

## 2. Materials and Methods

### 2.1. Materials

The study utilized SU-8 (Bisphenol A Novolac epoxy oligomer) provided by Gersteltech SA (Pully, VD, Switzerland), selected for its notable properties and applications in microfabrication, as well as for optical applications (refractive index and Absorption Coefficient of the utilized material can be found in Supplementary Materials Figure S6). The SU-8 came as dry pellets without any photoinitiator or additives. Cyclopentanone (99.0%, Sigma-Aldrich, St, Louis, MO, USA) was chosen as the solvent for preparing the solution electrowriting (SEW) inks due to its effective solubility with SU-8 and demonstrated workability in SU-8 electrospinning [30,31]. For the PDMS substrate and waveguide embeddings, Sylgard 184 silicone base and Sylgard curing agent (10:1) were utilized. The electrowriting setup was custom built and comprised nozzles provided by Nordson Engineered Fluid Dispensing(East Providence, RI, USA) (25 GA with 0.010 in (0.254 mm) tip diameter and 0.5 in (12.5 mm) tip length), a controlled XY stage from Newport Co. (Irvine, CA, USA) (managed via interfaced software), and a DinoLite Camera (AnMo Electronics Corporation, New Taipei City, Taiwan) for monitoring the Taylor cone during printing. The substrates used were clean glass slides (Silica, 26 mm × 76 mm × 1 mm) and float glass wafers, facilitating a variety of construct analyses. To obtain a cladding around the fibers, the printed filaments were deposited onto thin layers of PDMS spin-coated onto silica wafers. For MEW, SU-8 pellets were directly introduced into the syringe reservoir and utilized without modification. In contrast, for SEW, a solution was prepared by dissolving 75% (*wt.*/*wt.*) SU-8 pellets in 25% (*wt.*/*wt.*) cyclopentanone, followed by intense magnetic stirring overnight to ensure complete dissolution. This solution was then heated in an oil bath in sealed flasks at 100 °C for durations ranging from 30 to 75 min to induce partial crosslinking, enhancing the solution’s viscosity for electrowriting. Once prepared, the solution was transferred to the syringe reservoir and cooled to room temperature for at least 60 min before use. Utilized materials are summarized in Table 1.

### 2.2. Electrowriting Setup

**Figure 1:**
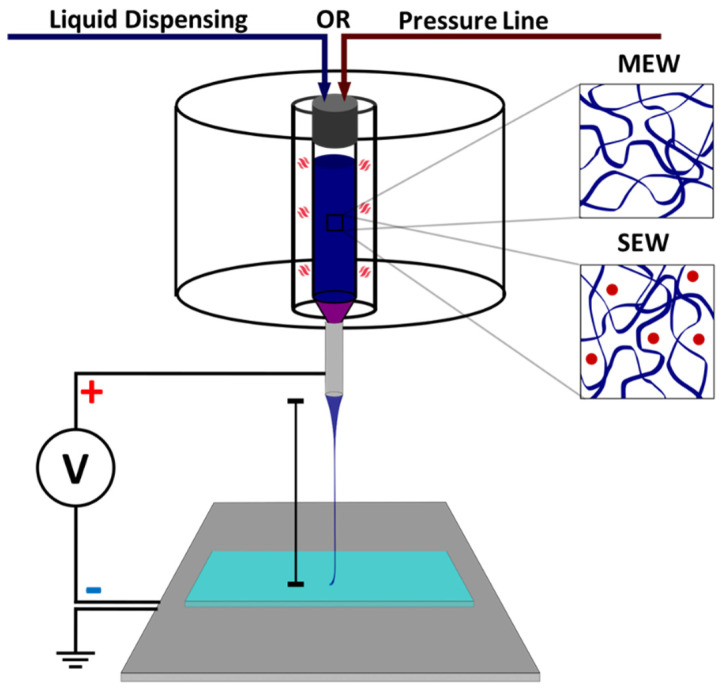
Setup utilized for MEW and SEW.

The electrowriting setup employed for MEW and SEW was engineered to enable the precise fabrication of polymer constructs through EHD processing. Central to the system is the application of voltage, within a range of 0.1–15 kV, supplied by a high-voltage source directly to a conductive aluminum plate, as depicted in Figure 1. This voltage plays a crucial role in the electrodynamic stabilization of the polymer jet, resulting in the formation of a Taylor cone. This phenomenon forms a droplet at the nozzle tip, yielding a continuous fiber of reduced diameter. The setup can process two types of materials: a polymer melt for MEW, which is heated above its melting point to form a viscous liquid, and for SEW, a polymer solution with solvated polymer chains, which also behaves as a viscous liquid suitable for fiber formation. The key to the process is the formation of a jet, where a continuous polymer filament is propelled from the nozzle tip to the receiver substrate, critically influencing the morphology of the resulting constructs. For MEW, jet formation depends on the cooling and solidification of the polymer, whereas SEW relies on solvent evaporation during the jet’s flight for precise polymer deposition. Parameters such as pressure, which sets the mass flow rate, and nozzle geometry influence fiber size and Taylor cone stability [15]. Other important components include the stage translation speed, which determines the nozzle-to-collector stage velocity, and the working distance, which is essential for maintaining the electrical field strength necessary for continuous fiber generation. Completing the system is a conductive plate stage that closes the circuit by applying a negative potential and a reservoir that holds the printable material, which is designed for compatibility with precision liquid dispensing. Surrounding the reservoir, a heating control mechanism uses resistances to accurately adjust the material temperature.

The key parameters for the MEW/SEW device operation visually illustrated in Figure 1 are the following: Voltage application for the electrohydrodynamic stabilization, which forms a Taylor cone upon stabilization, then thinning to microfiber and is collected in the receiver substrate, which provides adhesion of printed constructs (listed substrates in Materials section and Table 1). Working material: melt electrowriting uses pure polymer melt (illustrated by the blue polymer chains), and solution electrowritring uses polymer and solvent (blue chains = polymer + red dots = solvent). Pressure: mass flow rate of dispensed material. Nozzle: standard fluid dispensing metal nozzle that closes the voltage rig and provides the desired electrohydrodynamic stabilization. Stage: conductive aluminum plate moved in velocities in the mm × s range. Working distance: separation between nozzle and stage in the z-axis. Receiver substrate: provides adhesion of printed constructs and influences charge distribution of the printed jet. Material reservoir: a vessel that contains the printable material in a solid or liquid state, equipped with temperature control for MEW operation.

### 2.3. Printing Regimes, PDMS Substrate Embedding and Characterization Techniques

The printing process for both MEW and SEW involved setting the material (SU-8 melt or solution) in the reservoir at a specific temperature (above 110 °C for MEW, 25 °C for SEW). Key printing parameters, such as working distance (3–8 mm), stabilizing voltage (1–15 kV), and pressure (50 mbar–1 bar), were optimized for each regime. The initiation of the printing process was marked by a Taylor cone’s formation, after which the target pattern printing started.

For the MEW-waveguides embedded on PDMS, Sylgard 184 silicone base and Sylgard curing agent (10:1) ratio were mixed at 2000 rpm, then spin-coated into a Si-6inch wafer with a recipe for 300 µm thickness. The substrate was then left to homogenize for 15 min at atmospheric pressure. Posteriorly, the PDMS substrate was degassed for 1 h in a vacuum desiccator. Then, the PDMS substrate was placed in a petri dish and cured at 80 °C for 2 h. After the PDMS-coated substrate was cooled again to 25 °C, electrowriting of SU-8 can be performed directly on top of it. For the upper layer that completes the embedding, Sylgard 184 silicone base and Sylgard curing agent (10:1) ratio were mixed at 2000 rpm and poured directly over the printed SU-8 structures. Spin-coating was avoided at this point to avoid potential delamination of the SU-8 structures due to centrifugal force. The homogenization, degassing and curing steps were carried out in the same manner as the pristine substrate. The PDMS can be removed from the Si wafer with careful manual cutting and delamination with the aid of a scalpel.

Characterization of the printed constructs employed a combination of brightfield and darkfield microscopy (KEYENCE VHX-5000 optical digital microscope, KEYENCE, Itasca, IL, USA) and scanning electron microscopy (SEM MERLIN with a GEMINI II column at 3 kV acceleration voltage after a 10 nm gold sputtering). These methods provided detailed insights into the constructs’ morphology, structure, and potential optical properties. Image analysis for precise measurements of fiber diameters and construct geometry was performed using ImageJ software (Version 1.52a), complemented by the GIFT plugin [32,33].

## 3. Results

Initial experiments conducted with MEW on SU-8 polymer involved overnight heating at 70 °C, followed by an increase in temperature to 120 °C, and a 30 min wait for temperature stabilization prior to printing. These procedures yielded highly variable results, with fiber diameter expectedly decreasing with increased stage speed but lacking a discernible trend. This variability is attributed to the nature of SU-8, an epoxy-based resin that undergoes crosslinking with prolonged heat exposure, leading to changes in viscosity and overall rheological behavior that changes with the processing temperature ranges [13,14]. Consequently, a stable Taylor cone was challenging to achieve, resulting in frequent pulsing and the formation of highly tapered fibers (See Supplementary Materials Figure S1).

### 3.1. Melt Electrowriting: Shock Heat Protocol for SU-8

**Figure 2:**
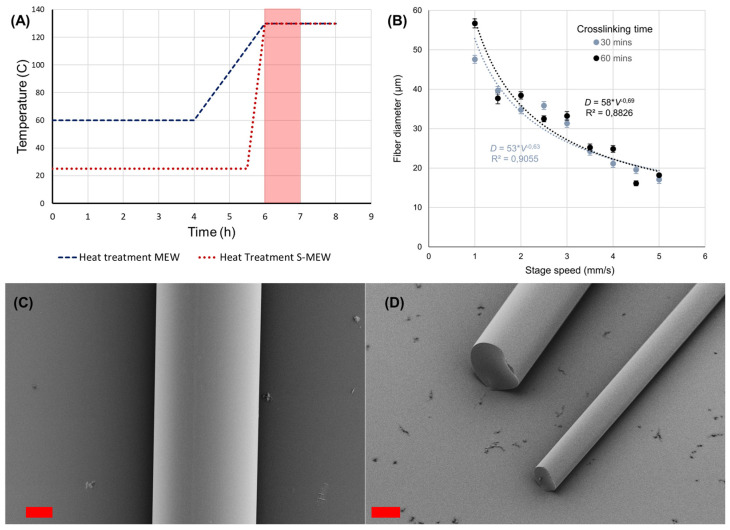
Protocol illustration: fiber diameter vs. stage speed and fiber morphology obtained for shock heat protocol MEW of SU-8. Operation conditions: voltage: 5 kV; shock temperature: 130 °C; operation distance: 3 mm; stage speed 0–5 mm/s; feedstock pressure: 500 mbar. Scalebar: 10 µm. (**A**) Heating protocol comparison for MEW vs. shock-heat MEW. (**B**) Fiber Diameter as a function of Stage speed for shock-heat MEW. (**C**) SEM micrography of the surface of a SU-8 Microfiber. (**D**) SEM micrography of cut ends of SU-8 Microfibers.

Given the limitations observed with the conventional MEW approach and considering the heat sensitivity of SU-8, a specific printing protocol dubbed ‘Shock Heat’ was devised. This method entails intensive heating at temperatures above the glass transition temperature (Tg ~60 °C) of low quantities (0.2–0.5 g) of uncured SU-8, setting the heating element at specific temperatures (120, 130, and 140 °C) and commencing printing after predetermined intervals (30, 60, 90 min) (an illustration for this heat treatment can be seen in Figure 2A). This technique aims to melt the polymer while controlling the extent of crosslinking, thereby facilitating printing after a short period of intensive heating, thus the ‘shock’ term. Fibers fabricated using this protocol exhibited consistent diameters, with a noticeable trend of diameter reduction associated with increased stage speed velocity, as is the rule with MEW-based techniques. This phenomenon reflects the real-time crosslinking impact on melt viscosity, which could complicate printing sessions longer than 60 min as fiber diameter changes over time. The results are summarised in Figure 2, for which stable operation MEW was achieved with SU-8 after 30 and 60 min of heating at 130 °C. After 90 min, the material’s rheological behavior turned unstable to print, showing frequent fiber pulsing [34] and irregular fiber diameter variance independent of stage translation speed. This is consistent with limitations found for MEW and the reported non-Newtonian properties of SU-8 when processed in melts [14,35]. Moreover, this approach allows for the processability of a thermoset polymer via MEW, which has not been reported in the past, as MEW is primarily dominated by thermoplastic polymers with low melting points [18]. Printing intricate constructs with long print times could be achieved by renewing the material and starting the heat shock treatment again. This approach could be further applied to other types of thermoset materials for which windows of stable operation can be identified, such as what is reported in this study, specifically for SU-8.

Figure 2 details print results after 30 and 60 min of printing. Figure 2A shows a heat cycling comparison of conventional MEW (blue dashes) vs. shock heat—MEW (red dots). Standard practices for MEW [4] usually operate by overnight heating at temperatures lower than printing T (60 °C) followed by a gradual increase in temperature, after which the printing session is started. For shock heat protocol MEW, the heat treatment to melt the polymer is applied in a much shorter time window (30 min), directly from room temperature (25 °C) to printing temperature (130 °C). This intense shock heat treatment allows for a manageable amount of crosslinking and a short print window (highlighted in red, duration = 60 min) for which controllable MEW printing can be achieved before the onset of crosslinking causes the print conditions to deteriorate and material needs to be renovated for controllable printing. Figure 2B shows the established relation between stage speed and average fiber diameter for the shock MEW regime for 30 and 60 min (*n* = 3 measurements along a fiber). Error bars represent standard deviations. Figure 2C Illustrates the characteristic smooth surface for SU-8 fiber printed by MEW (scalebar: 10 μm). Figure 2D shows the characteristic cross-section and cylindrical shapes of SU-8 fibers printed by S-MEW and then cut by a razor. These results show constant diameter and a smooth texture, as evidenced by SEM Microscopy, which is promising for photonic applications and waveguiding purposes [28,29] and is further developed in Section 3.3. Additionally, MEW fibers differ from SEW in the way they stack after being deposited one after the other due to the fibers being completely solid at their moment of deposition (see Supplementary Materials Figure S2).

### 3.2. Solution Electrowriting

The investigation into SU-8’s solution electrowriting began with formulations known to facilitate electrospinning [30,31], marking the first time applying these formulations to SEW. The process involved using a 75% *wt.*/*wt.* SU-8 solution, exploring various printing protocols. Initial attempts highlighted the solution’s inadequate viscosity for SEW, leading to outcomes more akin to electrospraying, characterized by the dispersion of discrete particles and the formation of thin fibers indicative of a whipping behavior typical of electrospinning [8] (see Supplementary Materials Figure S3). To address this, the solution underwent heat-induced partial crosslinking to lengthen polymer chains, thus enhancing the solution’s viscosity and altering the Taylor cone’s behavior and droplet composition. This modification significantly impacted the fluid’s behavior and printability, attributed not just to changes in viscosity but also to the solution’s elasticity—its capacity to stretch without breaking—critical for maintaining stable printing regimes.

Five formulations with varying durations of partial crosslinking were developed for testing. These were submitted to heating at 100 °C for 30, 45, 60, 75 and 90 min. Among these, SU-8 solutions crosslinked for 75 and 90 min proved excessively viscous, hindering its flow and causing it to adhere strongly to the flask and syringe reservoir walls, rendering it impractical for use. Solutions heated for 30 and 45 min were insufficiently viscous, leading to jet breakage and whipping behavior such as observed in Supplementary Materials Figure S2. The exploration of the parameter space available reveals that partial crosslinking markedly affects both the fluid dynamics and printability of the inks. This influence extends beyond viscosity alterations to affect the solution’s elasticity, which is pivotal for the jet’s ability to stretch without severing, a critical factor for achieving consistent printing regimes [36,37].

The ink that was crosslinked for 60 min was chosen for further analysis given its shown stability upon variation in pressure, voltage, and stage translation speed. (For more information on the ink comparison and testing, see Supplementary Materials Figure S4.)

**Figure 3:**
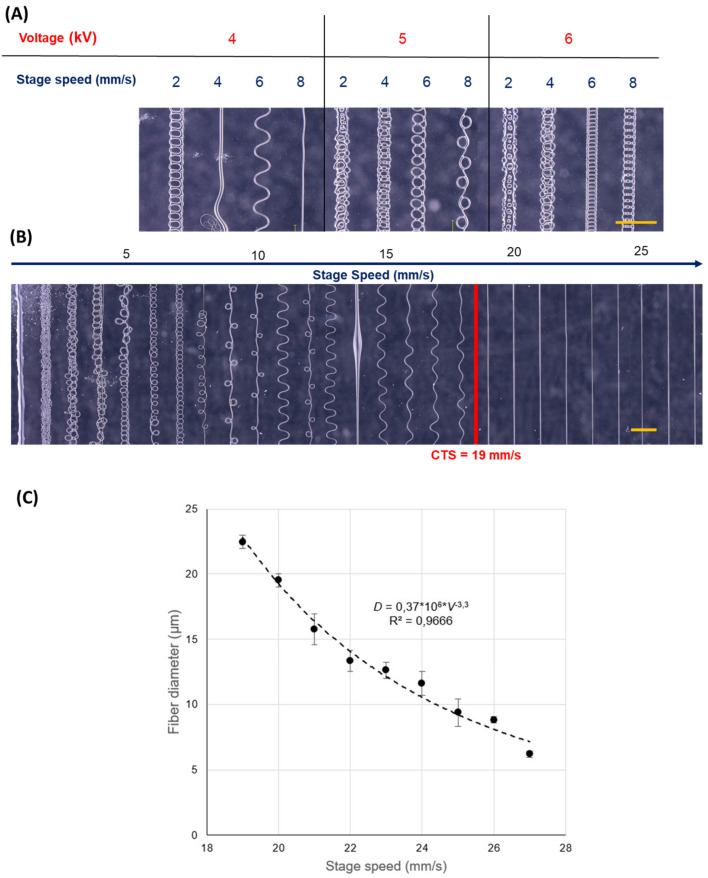
Parameter screening for 60 min crosslink ink. Scalebar: 500 µm. Stage distance of 3 mm and pressure inlet of 500 mbar was kept constant for all runs. (**A**) Comparison of voltage effect. (**B**) Critical Translation Speed (CTS) determination, marked by a red line at 19 mm/s. Voltage for this run was fixed at 5 kV. (**C**) Fiber diameter as a function of stage speed from the fibers shown in (**B**).

Figure 3 illustrates the evolution of fiber deposition under specified conditions up to the Critical Translation Speed (CTS) of 19 mm/s, at which point straight fibers are produced. The observed progression mirrors that seen in other speed sweep experiments, showcasing consistent jet behavior trends. Initially, conjoined structures emerge as the jet folds into itself at speeds ranging from 1 to 5 mm/s. Following this, the formation of coils occurs between 6 and 18 mm/s, where, despite the coiling, a uniform fiber diameter can be discerned. Within this speed regime, specific velocities also give rise to sinusoidal patterns, frequently observed in MEW experiments conducted below the CTS [38]. These sinusoidal formations may alternate with coiling patterns as the jet undergoes lower-frequency rotations prior to reaching the collector surface. Furthermore, the amplitude of sinusoidal patterns is seen to increase until they flatten out entirely, culminating in the formation of straight fibers. The subsequent plot details the relationship between the fiber diameter and the stage speed. The resulting fiber diameter decreases with stage speed, as expected, showing a velocity-dependent decline starting from the Critical Translation speed of 19 mm/s, the initial diameter given by the pressure parameter, and adjustable by increasing the operation velocity. For stage velocity of 14 mm/s, a ‘pulsing’ behavior is shown, which is a common occurrence and unsolved problem characteristic of EW-based techniques, which may appear during prolonged operation due to force imbalances leading to mass flow oscillations and thus these pulsing beads [34].

**Figure 4:**
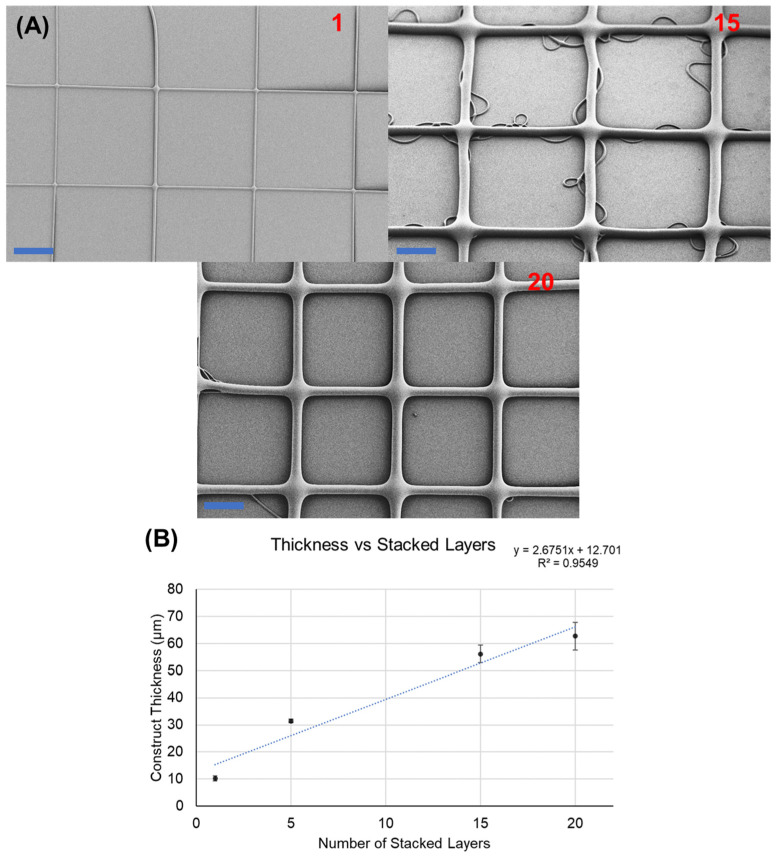
Three-dimensional structures and fiber merging in SEW. Print conditions: ink 60 min crosslinking; working distance: 3 mm; pressure inlet: 350 mbar; voltage: 5 kV; stage speed: 30 mm/s. (**A**) Fiber merging in multi-layered constructs as seen in scanning electron microscopy. Scalebar: 200 µm. Numerals in red indicate the number of layers. (**B**) Construct thickness increases per number of layers from SEM images.

Extensive characterization of operational parameters facilitated the testing of SEW for multi-layered construct fabrication. Constructs demonstrated fiber merging at intersections and overlays, increasing construct width and height, and eliminating visible stacking evidence, as depicted in Figure 4 for SEW and Supplementary Figure S2 for MEW of SU-8. This merging behavior has been shown to appear before on electrospinning jets [39]. This phenomenon is thought to originate from the effect of surface tension; thus, merging arises if the reduction in surface energy due to merging is lower than the local increase in the electric field in the extruded jet. The result is that the layers are indistinguishable from each other as in regular MEW or techniques like Fused Deposition Modeling (FDM) [40]. The increase in height and width was analyzed, and it is reported in Figure 4.

### 3.3. Optical Guiding Demonstration

**Figure 5:**
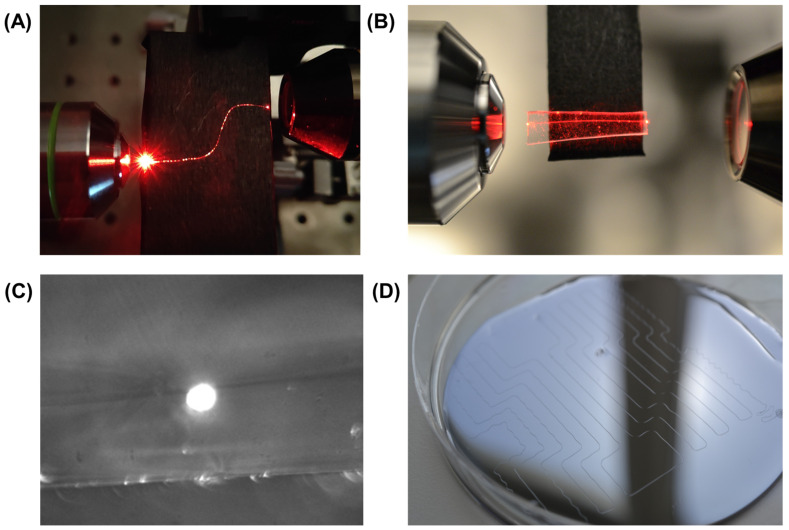
Optical guiding in SU-8 fibers with and without PDMS cladding: (**A**) Light coupling within SU-8 Fiber employing a He-Ne laser. (**B**) Light coupling within SU-8 Fiber with incorporated PDMS cladding employing a He-Ne laser. Noticeable scattering is still observed in both cases. (**C**) Output facet of the SU-8 fiber encased in PDMS cladding, revealing the illuminated core of the fiber and the distict boundary between the PDMS bottom and top layer. (**D**) Top view of the SU-8 fiber encased in PDMS cladding.

In this section, the optical guiding of SU-8 fibers fabricated using shock heat protocol MEW was probed due to its optimal fiber surfaces, as well as the larger fiber dimension that facilitates the light coupling into the fiber core. In Figure 5, a demonstration is presented: showing light coupling employing a 633 nm He-Ne laser (Figure 5A). Light guidance within the waveguides is observed through scattering along the fibers, as viewed from the top. Due to the absence of cladding and the direct contact of the waveguides with the substrate, significant scattering occurs, highlighting the light path through the fiber. To circumvent this scattering, Polydimethylsiloxane (PDMS) was implemented as a substrate cladding material (Figure 5B). This methodology entails an initial step where a layer of PDMS is spin-coated onto the substrate to serve as the bottom cladding layer (preparation described in the Materials section). Subsequently, SU-8 fibers are directly printed onto this layer using shock heat protocol MEW. Following the printing process, a second PDMS layer is deposited over the fibers, thus completing the cladding structure as depicted in Figure 5C. This approach not only promises to reduce scattering losses by preventing direct contact between the waveguides and the substrate but also offers a method to finely tune the waveguide’s optical properties through the manipulation of the cladding refractive index. The resulting speckle images from the fiber output facet, as well as the basic optical setup utilized for the coupling, can be found in Supplementary Materials Figure S7. Moreover, employing PDMS as cladding material could introduce flexibility to the waveguides, expanding their application potential in flexible electronics and wearable photonics, where robustness to mechanical stress is paramount (See Supplementary Materials Figure S5). Qualitatively, while scattering is reduced, it remains notably present. Figure 5C displays the output facet of the fiber with white light coupling to enhance visibility. Along with the core area, the boundary between the two PDMS layers is distinctly visible, suggesting that the bottom and top PDMS coatings did not merge effectively, likely resulting in air gaps between them. These air gaps can act as significant scattering centers. In Figure 5D, the top view of the SU-8 fiber with PDMS cladding is presented.

## 4. Discussion

In comparing the two electrowriting methods for SU-8, significant differences emerge in terms of operational parameters and the characteristics of the resulting constructs. Despite both methods yielding smooth fibers or constructs, as evident from SEM imagery, the intricacies of their processing and outcomes reveal distinct advantages and challenges. For instance, the bending of MEW fibers exhibits a radius that is influenced by the viscoelastic properties of the jet, leading to bends that occur with varying sharpness based on material behavior [41]. SEW, on the other hand, demonstrates more precisely defined corners, particularly at 90° bends, allowing for more accurate deposition in varied pattern regimes. Notably, SEW exhibits a capability for quicker transitions between different printing regimes, as evidenced by the immediate pattern changes following velocity adjustments post-bend.

MEW offers a method to produce straight waveguides with minimal diameter variability, presenting an advantage for applications requiring uniformity in structural dimensions. However, MEW encounters challenges during prolonged printing operations due to viscosity variations in the melt over time. Conversely, SEW introduces the ability to modulate viscosity through the degree of crosslinking within the SU-8 solutions. Both SEW and MEW could benefit from further analysis, such as Fourier Transform Infrared Spectroscopy to quantify crosslinking bonds [42,43] and rheological measurements to determine solution viscosity, be it in solution or to further characterize the printable window for thermoset SU-8. Although working with a solution introduces complexities related to charge interactions within the electrowriting setup and the behavior of different chemical species under printing conditions, the potential for achieving diverse construct properties through SEW presents a compelling tradeoff, especially in the case for processability and access to thermoset polymers. For the case of SEW, the ability to fine-tune material properties via solution-based methods opens new avenues for the development of constructs with tailored functionalities, balancing the operational challenges with the promise of expanded application potentials. Moreover, fiber merging behavior in SEW suggests a novel approach to fabricating 3D microstructures by exploiting fiber merging, potentially enabling the creation of complex, monolithic constructs with tailored microstructural features and integrated functionalities, such as engineered refractive indices studied in the literature with two-photon polymerization. The fiber merging phenomenon, similar to natural structural materials like bone or shell, suggests a seamless integration at joints, offering new possibilities in microfabrication and design spaces for optical applications, including seamless waveguide joining. This comprehensive exploration of MEW and SEW for SU-8 underscores the potential of these techniques in advancing the capabilities of additive manufacturing technology for high-resolution, functional 3D constructs [44,45,46,47].

## 5. Conclusions

In this study, an exploration of the potential of electrowriting for fabricating SU-8 constructs was performed using both MEW and SEW.

What was achieved can be summarized in the following points:

Melt electrowriting of SU-8 microfibers:

- Shock heat protocol devised for SU-8. Intensive heating for a short amount of time (30 min before printing) allows the processing of an SU-8 material. This could be further extended to other heat-sensitive materials or thermoset polymers and resins compatible with MEW.
- MEW-based printing of smooth-textured fibers suitable for photonic and waveguiding applications. Obtention of fibers from 60 to 10 µm diameter range.
- Validation of printing directly on PDMS substrates and further embedding with a second PDMS layer, which can be implemented to provide fibers with flexible cladding for photonic device fabrication.

Solution electrowriting of SU-8 microfibers and 3D constructs:

- SEW printing utilizing 75% wt. SU-8 ink, resulting in the repeatable obtention of microfibers with a diameter size ranging from 25 to 5 µm.
- Extensive characterization of the parameters utilized for printing. Voltage (kV), pressure (mbar range), stage speed, crosslinking times, and their influence on the printability of SU-8.
- Strategy to increment ink viscosity via in-solution material crosslinking.
- Obtention of 3D constructs that show microscale fiber merging. Coupled with AM principles, this suggests the potential for functional multilayer constructs with localized control over physicochemical properties by incorporating nanomaterials into the SU-8 base.

This investigation opens new processing possibilities within AM for SU-8, building upon existing knowledge of this important optical material. The integration of SU-8 processing expertise, derived from conventional CMOS methodologies, with these novel AM techniques, offers exciting prospects for innovative device designs and architectures. Future work could further enhance this field by quantitative analysis of the optical coupling efficiency and losses in waveguides and photonic components created via electrowriting. This work potentially provides a cost-effective alternative to waveguide fabrication of photonic systems using direct laser writing.

## Figures and Tables

**Table 1:** Materials utilized for MEW and SEW.

	Shock Heat Protocol Melt Electrowriting (MEW)	Solution Electrowriting (SEW)
**Printing Materials**	SU-8 pellets (solid, uncured)	SU-8 (25% *wt.*/*wt.* solid, uncured) + cyclopentanone (75% *wt.*/*wt.*)
**Substrates**	Glass slides (Silica, 26 mm × 76 mm × 1 mm) 6-inch float wafers (SiO_2_ glass) 6-inch Si wafer + PDMS coat	Glass slides (Silica, 26 mm × 76 mm × 1 mm)

## Data Availability

The data presented is contained within the article and Supplementary Materials. The raw data supporting the conclusions and calculation will be made available by the authors on request. Additional imaging or processing data are available upon reasonable request to diego.sandovalsalaiza@epfl.ch due to potential conflicts of interest with ongoing investigations.

## Supplementary Materials

The following supporting information can be downloaded at: https://www.mdpi.com/article/10.3390/polym16121630/s1, Figure S1. Tapered Fibers obtained using conventional MEW with SU-8; Figure S2. Stacked fibers characteristic from MEW; Figure S3. Electrospraying-like operation for SEW of SU-8; Figure S4. Multiple parameter screening for 30, 45, and 60 min crosslinked SU-8 ink in SEW operation; Figure S5. SU-8 fibers embedded in a flexible PDMS substrate; Figure S6. Absorption coefficient and refractive index of SU-8 as measured by ellipsometry. Adapted from data obtained from [48]. Figure S7. Basic optical characterization setup and readout from optical fiber output.
