# Supplementary figures and images for: Electrowriting of SU-8 Microfibers
Source: Polymers (Basel). 2024 Jun 8;16(12):1630. doi: 10.3390/polym16121630 (PMC11207615; doi:10.3390/polym16121630)

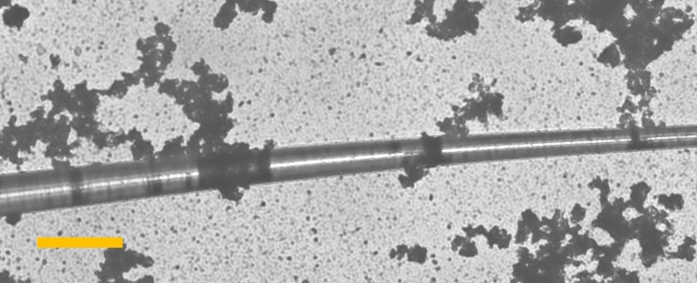

Supplement: Supplementary file 1 [file polymers-16-01630-s001.zip › Figure S1. Fiber by normal MEW.png]

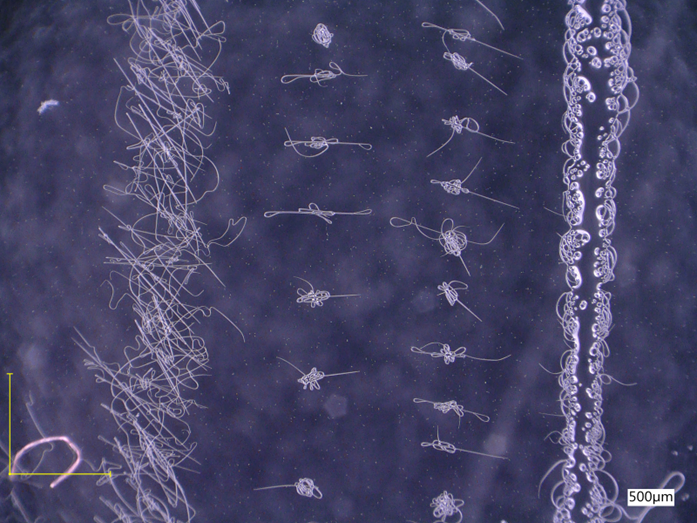

Supplement: Supplementary file 1 [file polymers-16-01630-s001.zip › Figure S3. Electrospraying regime..png]

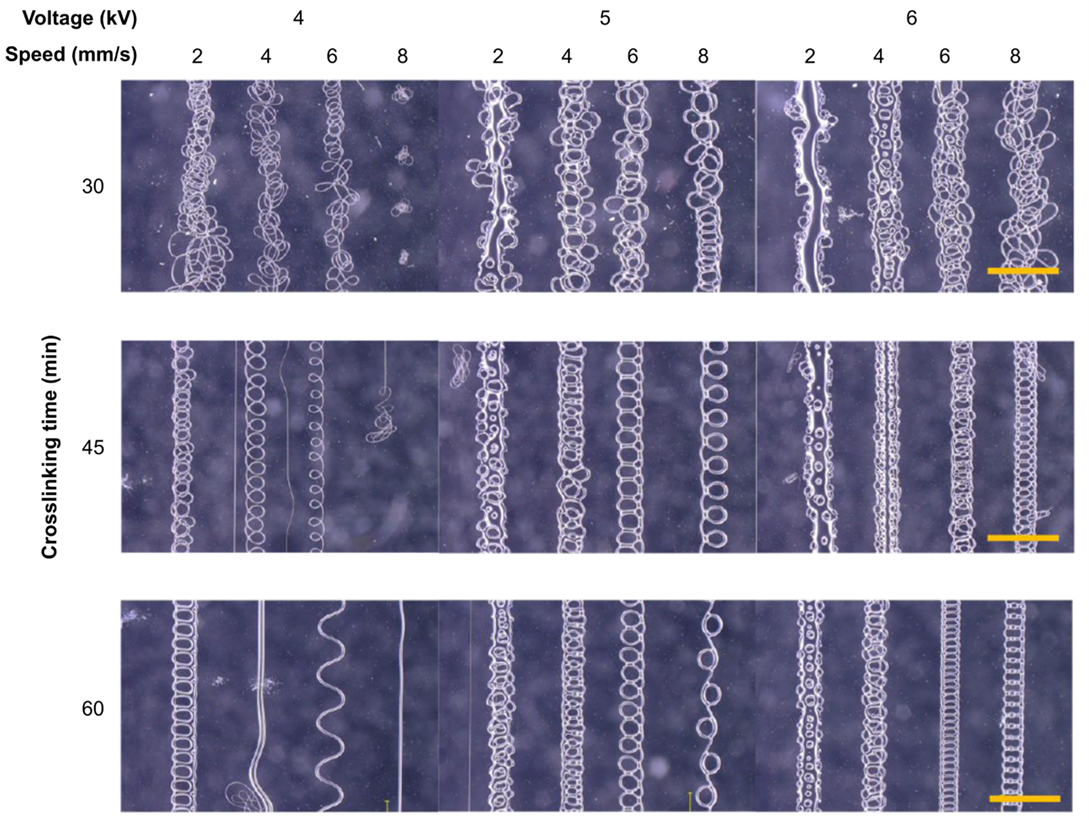

Supplement: Supplementary file 1 [file polymers-16-01630-s001.zip › Figure S4. Parameter Exploration of SU8 by SEW.png]

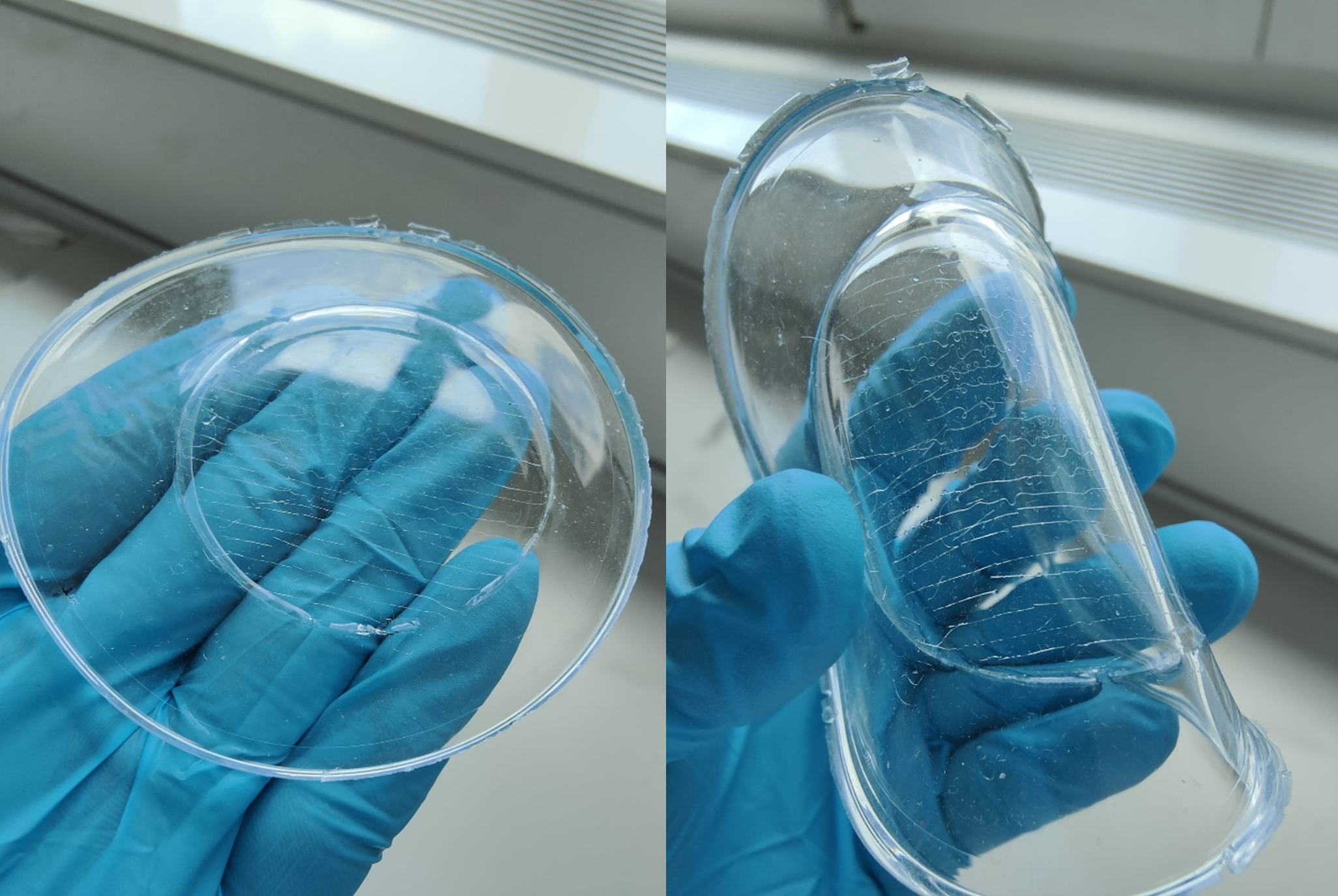

Supplement: Supplementary file 1 [file polymers-16-01630-s001.zip › Figure S5. SU8 Fibers embedded on PDMS.png]

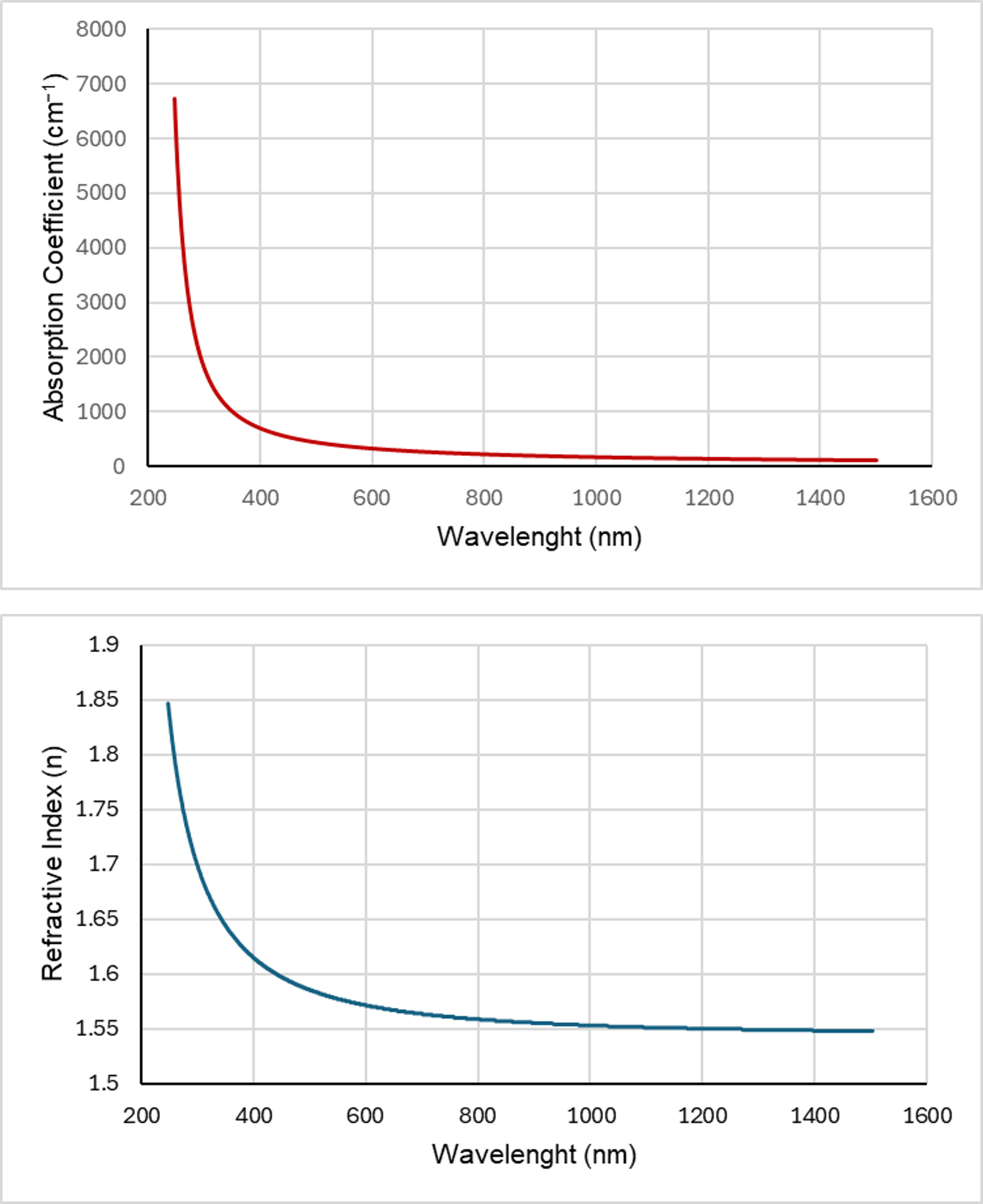

Supplement: Supplementary file 1 [file polymers-16-01630-s001.zip › Figure S6. RefractiveIndex_Absorption.png]

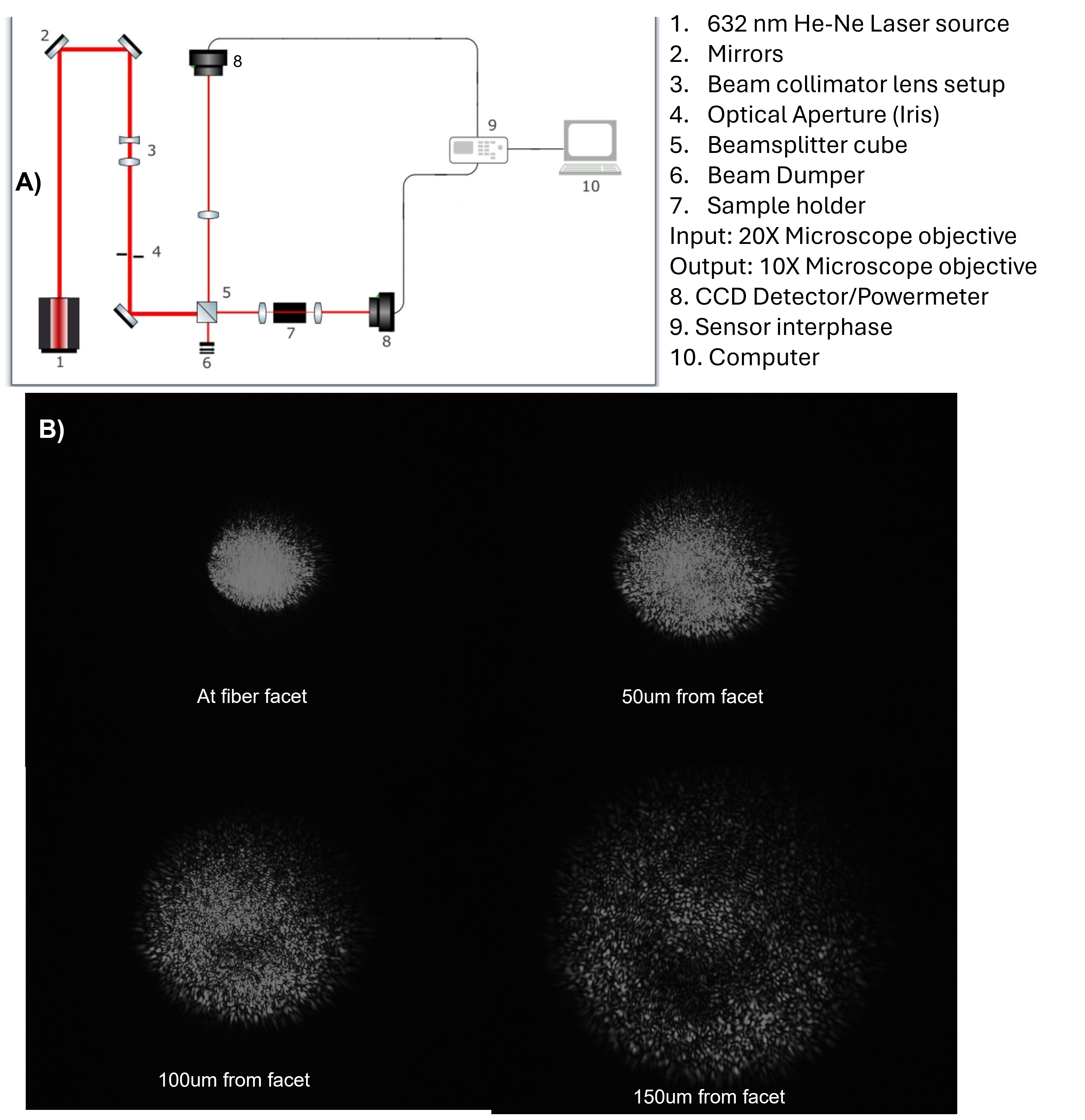

Supplement: Supplementary file 1 [file polymers-16-01630-s001.zip › Figure S7. Optics.png]
